# The impact of a 12-hour educational program on nurses’ knowledge and attitudes regarding pain management: a quasi-experimental study

**DOI:** 10.1186/s12912-022-01028-4

**Published:** 2022-09-07

**Authors:** Adnan Innab, Kamila Alammar, Naji Alqahtani, Fatima Aldawood, Ali Kerari, Ali Alenezi

**Affiliations:** 1 Nursing Administration and Education Department, College of Nursing, King Saud University, Riyadh, 11421 Saudi Arabia; 2 Anfas Medical Care Hospital, Riyadh, Saudi Arabia; 3 Ministry of National Guard Health Affairs, Riyadh, Saudi Arabia; 4 Medical Surgical Nursing Department, College of Nursing, King Saud University, Riyadh, Saudi Arabia; 5 King Fahad Hospital in Madinah, Riyadh, Saudi Arabia

**Keywords:** Pain, Sedation, Knowledge, Attitude, Nurses, Education, Self-efficacy, Satisfaction, Saudi Arabia

## Abstract

**Background:**

Proper pain assessment is fundamental to effective pain management. Training nursing staff is critical for improving *pain assessment competence and patient clinical outcomes.* However, there is a dearth of research examining interventions that can enhance nurses’ knowledge and attitudes toward pain management, especially in Saudi Arabia. Thus, this study aimed to evaluate the effectiveness of a structured education program on nurses’ knowledge and attitudes towards pain management.

**Methods:**

A quasi-experimental design was used. The study sample included 124 registered nurses working in intensive care or inpatient units in Saudi Arabia. Data were collected between March and September 2021 using a knowledge and attitudes survey regarding pain, satisfaction with and self-confidence in learning, and the learning self-efficacy scale for clinical skills.

**Results:**

Nurses showed moderate levels of knowledge and attitudes regarding pain before (M = 20.3, SD = 4.80) pain management education, which were significantly higher after the intervention (M = 22.2, SD = 5.09, *t* = 2.87, *p* < .01). Before the intervention, nurses with a baccalaureate degree had more knowledge and better attitudes regarding pain management than diploma nurses (*t* = 3.06, *p* < .01). However, there was no significant difference between the two groups after the intervention (*p* > .05), indicating that the education was effective in enhancing nurses’ knowledge and attitudes, regardless of nursing education level. Nurses in this study had high mean scores for self-confidence in learning (M = 35.6, SD = 4.68, range = 18–40), self-learning efficacy (M = 52.9, SD = 7.70, range = 25–60), and satisfaction with learning (M = 22.2, SD = 3.24, range: 10–25).

**Conclusion:**

Regular pain education programs can improve nurses’ knowledge and attitudes. Increasing the breadth and depth of educational courses, alongside appropriate training, competency-based assessment, and pain education programs, is also recommended. Future research should consider the subjectivity and individualized nature of nursing by including patient satisfaction surveys to measure the improvement in nurses’ knowledge and attitudes from the patient perspective.

## Introduction

The International Association for the Study of Pain has defined pain as “an unpleasant sensory and emotional experience associated with, or resembling that associated with, actual or potential tissue damage” [1]. Pain affects all people, irrespective of their health status. According to the World Health Assembly, patients have the right to pain relief, and health professionals have an ethical duty to alleviate patients’ pain and suffering [2]. Additionally, the Joint Commission International has stressed the importance of pain assessment and management that is designed to meet patients’ needs [3]. Additionally, the World Health Organization has proposed an analgesic ladder strategy to provide adequate pain relief for patients through educational campaigns [4]. The ladder’s central tenet is the importance of acquiring appropriate knowledge to accurately assess and manage patients’ pain [5].

Inadequate pain assessment and management adversely affects patient outcomes, including prolonging the length of hospital stay and delaying patient recovery [6]. Nurses serve as patients’ advocates; thus, it is crucial for them to appropriately assess and manage patients’ perceptions of pain. However, studies have shown that nurses have inadequate knowledge and poor attitudes toward pain [6–8].

Various factors have been identified as barriers to effective pain assessment and management among nurses, including their lack of knowledge and skills, poor teamwork, high workload, lack of nurse-patient communication, and inadequate time [9–11]. Since one important factor that contributes to inadequate pain management is the lack of knowledge and attitudes toward pain among nurses, a pain management educational program could be an effective strategy for enhancing their knowledge and attitudes.

Learning reflects teaching quality; thus, effective teaching and learning strategies are a critical component of any educational program [12]. The use of simulation in educational programs has been found to boost learning experiences, leading to improved self-efficacy, learner satisfaction, and self-confidence [13, 14], and simulations have been used to advance healthcare providers’ diagnostic abilities as well as their motor and technical capabilities [15]. The expertise, self-confidence, and efficacy gained through learning are crucial aspects of providing high-quality care; therefore, nurses need to be equipped with the knowledge and skills to provide effective and appropriate assessment and management of pain [16, 17].

## Background

In 1996, the American Pain Society introduced pain as the fifth vital sign, along with the vital signs of temperature, respiratory rate, blood pressure, and pulse rate, emphasizing the importance of pain assessment [18]. The pathogenesis of pain sensation encompasses all mechanisms contributing to acute or chronic pain [19], and proper pain assessment enables effective management. Consequently, a lack of consistency and specificity in pain assessment may complicate pain management among ill patients [20].

Pain management is a responsibility of every healthcare professional and is pivotal responsibility for nurses [21]. Ineffective pain management results in a sizable reduction in desirable clinical and psychological outcomes, as well as the patient’s overall quality of life [22]. Research has focused on the barriers identified by nurses that affect proper pain management. These barriers include inadequate knowledge and lack of pain assessment skills, misconceptions, lack of time to assess and control pain, inability to speak for patients’ rights, and poor educational background [7, 20, 23]. Therefore, nurses’ pain management can be enhanced through continuous training, competency-based assessment, and pain education programs are necessary [24–26].

A confident nurse is a patient’s advocate, ensuring that the patient receives the best possible care [27]. When nurses have the necessary skills, they are confident in their assessment and management of patients’ pain [28]. Pain management training has been shown to influence nurses’ beliefs in their ability to manage pain [29]. Education is fundamental for improving healthcare professionals’ skills and building confidence [30]. Utilizing simulation fosters learning satisfaction and self-confidence among healthcare professionals [31, 32]. Self-efficacy is critical for modifying a person’s behavior [33], and self-confidence and satisfaction levels impact anxiety and self-efficacy in patient care [34].

Several observational studies have assessed nurses’ pain knowledge and attitudes [21, 35, 36]. However, to the best of our knowledge, there is limited research examining interventions to enhance nurses’ knowledge and attitudes toward pain management, especially in Saudi Arabia (SA). Thus, this study aimed to evaluate the effectiveness of a structured education program on nurses’ knowledge and attitudes toward pain management. The specific aims were: 1) to assess staff nurses’ knowledge and attitudes regarding pain before and after pain management education; 2) to examine the association between the sample characteristics and their knowledge and attitudes regarding pain, pre- and post-pain management education; and 3) to describe staff nurses’ self-confidence, self-efficacy, and satisfaction on learning post-pain management education.

## Methods

### Design and sample

A quasi-experimental pre- and post-test research design was utilized and followed the TREND checklist*.* The study sample comprised registered nurses working in intensive care units (ICUs) or inpatient units in long-term care (LTC) hospitals providing palliative and critical care. The study’s inclusion criteria were: 1) being a registered nurse; 2) having at least 6 months of experience working in an ICU or LTC unit; and 3) providing consent to participate in the pre-and post-test surveys. Nursing administrators, nurses working in outpatient clinics, and those unable to attend the two-week sessions were excluded from the study. Considering a significance level of .05, a power value of .80, and an effect size of .3, a minimum of 67 participants was needed for the statistical analyses. Overall,124 registered nurses were recruited as participants.

### Data collection procedure

Following approval of the study from the relevant Institutional Review Board, the study was conducted in three phases over 6 months (March–September 2021). In Phase 1, the baseline data were collected in which researchers obtained pre-test data from eligible nurses via a one-hour online survey sent to their hospital email addresses. In Phase 2, a 12-hour educational program was conducted that included a number of educational strategies. In Phase 3, the post-test was administered 1 month later as a one-hour online survey sent to the nurses’ email addresses. The nurses were instructed to complete the questionnaires independently.

### Research intervention

A nurse educator and clinical instructor who had more than 10 years of experience in nursing education, specifically in surgery, radiology, cardiology, and LTC, delivered the program. The program was administered in an LTC facility providing palliative and ICU patient care. The course was developed to cover the required procedural sedation and pain management topics to ensure that the nursing staff, who administered sedation, received education and training on moderate sedation that addressed proper pain assessment, reassessment, and management as required by the Saudi Central Board of Accreditation of Healthcare Institutions [37]. As part of the general nursing orientation and preparation for practice, newly recruited nurses must pass the pain assessment and reassessment competency. Since pain assessment training is required by long-term accreditation bodies and hospital policy, it is provided regularly.

The training program was conducted at a private hospital in SA that was well equipped with modern training equipment and an adequate number of auditoriums. This study involved a structured education program developed to improve nurses’ knowledge and attitudes toward pain management. The main objectives of the pain management educational program were: 1) to prepare nurses to care for patients receiving acute and chronic pain management; 2) to implement appropriate strategies and tools for pain assessment; and 3) to administer pharmacological and non-pharmacological approaches for pain management.

The 12-hour pain management educational program comprised four sessions that covered several topics related to pain management (Table 1): pain assessment and reassessment tools; barriers to effective pain management; pain management (pharmacological and non-pharmacological); patient-controlled analgesia; respiratory and cardiac complications; reversal agents; patient monitoring and documentation; speaking up for patients’ rights; and patient/family education. The teaching methods used in the educational program included face-to-face lectures, case studies, group discussions, and skill demonstrations using task trainer simulation. Each session included theory-based teaching and clinical practice that lasted around 2 to 4 hours. More educational hours were given to the session including hands-on skills demonstration using the task trainer simulation (e.g., B. Braun automated infusion pumps and Adult Airway Management Trainer: Airway Larry).

**Table 1:** Content included in pain management course

Course Content	Methodology	Hours
Session 1 •Pain Definition •Pain Physiology •Types of Pain	•Lecture •Group iscussion	•2 hours
Session 2 •Pain Assessment and Reassessment Tools •Pain Management (non-pharmacological and pharmacological, barriers to effective pain management, and patient-controlled analgesia)	•Group discussion •Hands-on activity •Demonstration and re-demonstration (B. Braun automated infusion pumps)	•4 hours
Session 3 •Respiratory and Cardiac complications •Reversal Agents	•Lecture •Case study •Adult Airway Management Trainer (Airway Larry)	•4 hours
Session 4 •Patient Monitoring and Documentation •Speaking Up for Patients’ Rights •Patient/Family Education	•Lecture •Group discussion •Case study	•2 hours

### Measures

Sociodemographic information included age, sex, level of education, general length of experience in nursing, length of experience in the current unit, and previous training in pain management. The Knowledge and Attitudes Survey Regarding Pain (KASRP), satisfaction with and self-confidence in learning, and Learning Self-Efficacy Scale (L-SES) for clinical skills were used in this study. All instruments were used without any modifications.

#### Knowledge and attitudes survey regarding pain

The KASRP was originally developed to reflect changes in pain management practices among nurses and other healthcare professionals [38]. It can be used to evaluate nurses’ knowledge and attitudes regarding pain in pre- and post-educational programs [7, 25]. The survey covers areas of pain management using either pharmacological or non-pharmacological approaches and comprises 39 items. Fifteen items are multiple-choice questions, 22 are True/False statements, and two are case studies with multiple-choice options. Each correct answer was assigned one point, with total scores ranging from 0 to 39. A higher score indicated greater knowledge and attitude toward pain management. Content validity was established by a panel of subject matter experts, whereas construct validity was established by comparing the responses of nursing students, staff nurses, and experts in pain management [38].

#### Satisfaction and self-confidence in learning

The National League of Nursing developed this self-report Satisfaction and Self-Confidence in Learning scale to evaluate learners’ simulation-based experiences [39]. It consists of two subscales: satisfaction with current learning and self-confidence in learning. Thirteen items are measured on a 5-point Likert scale, ranging from *strongly disagree* to *strongly agree.* The Cronbach’s alpha of the subscales ranged from .87 for the self-confidence in learning subscale to .94 for the satisfaction subscale [39, 40]. To our knowledge, this instrument has previously been used with samples of nursing students, but not with nurses working in acute care settings.

#### Self-efficacy scale for clinical skills

The Self-Efficacy Scale for Clinical Skills was used to measure nurses’ self-efficacy with their clinical skills. It consists of 12 items on three domains: cognitive, affective, and psychomotor skills [41]. Items were measured on a 5-point Likert scale, ranging from *disagree* to *agree*. In this study, the Cronbach’s alpha for this scale was .96. The content validity index for all the questions was between .88 and 1, indicating high content validity [41].

### Ethical considerations

The approval of the Institutional Review Board was obtained before data collection [KSU-HE-21-196], while the approval to use the instruments was obtained from the original authors. Participants were given a recruitment statement that contained the purpose of the study, risks and benefits, and confidentiality of their information. Nurses were informed that their participation was voluntary and that they could withdraw from the study at any time without any consequences. Nurses interested in participating were given the consent form to be signed two weeks before the intervention. No names or personal information were collected. However, each participant was given a particular code used in the pre- and post-intervention tests to match their responses. Participants were informed that the data would be reported in aggregate form.

### Data analysis

The Statistical Package for Social Sciences (SPSS) software V. 28 (IBM Corp., Armonk, NY, USA) was used for the data analysis. Descriptive statistics were calculated as frequencies and percentages for categorical variables (sex, level of nursing education, working unit, and previous pain management training) and measures of central tendency for continuous variables (years of experience, knowledge, and attitudes regarding pain, self-confidence, self-efficacy, and satisfaction).

For the first specific aim, paired-sample *t*-tests were performed to examine the mean differences in knowledge and attitudes regarding pain pre- and post-pain management education. For the second specific aim, Pearson’s correlations, independent sample *t*-tests, and one-way analysis of variance (ANOVA) were conducted. For the third specific aim, the means (M), standard deviations (SD), and range were calculated. The significance level was set at *p* < .05.

## Results

### Demographics’ characteristics

The participants’ sociodemographic characteristics are displayed in Table 2. The nurses in this study had an average of 6.2 years of experience (SD = 3.23). The majority of nurses were women (*n =* 113, 90.4%), held a diploma degree in nursing (*n =* 87, 69.6%), and provided care in general inpatient units (*n* = 79, 63.2%). More than half participants (*n =* 63, 50.4%) had not received previous training in pain management.

**Table 2:** Participants’ Sociodemographic Characteristics (*N* = 125)

Variable (Range)	***n*** (%) or M (SD)
**Sex**	
Male	12 (9.6)
Female	113 (90.4)
**Years of Experience in Nursing**	6.20 (3.23)
**Level of Nursing Education**	
Diploma	87 (69.6)
BSN	38 (30.4)
**Working Unit**	
Critical	37 (29.6)
General	79 (63.2)
Outpatient	9 (7.2)
**Previous Pain Management Training**	
Yes	62 (49.6)
No	63 (50.4)

Table 3 shows the percentages of correctly answered items on the questionnaire. In the pre-test, the highest proportion of correct responses was given on appropriate sedation assessment, the definition of narcotic/opioid addiction, and opioid analgesic administration. On the post-test, many respondents improved their performance on the KASRP. On average, participants answered 49.5% of the questionnaire items correctly before the intervention, increasing to 53.6% after the intervention. The highest proportion of correct answers corresponded to the items related to vital signs as pain indicators, patients’ spiritual beliefs, recommended route of administration for opioid analgesics, and the peak effect of IV morphine. However, some items such as the use of opioid medications and recommended opioid dose, appropriate pain assessment and diagnosis, and reliable indicators of pain intensity remained the same.

**Table 3:** Item Analysis of Knowledge and Attitudes Survey Regarding Pain Management

	No. Item Content	Correct Responses				***P***-value
		Pre-test		Post-test		
		N	%	N	%	
1.	Because their nervous systems are underdeveloped, children under 2 years of age have decreased pain sensitivity and limited memory of painful experiences	55	44	78	62.4	.002**
2.	Vital signs are always reliable indicators of the intensity of a patient’s pain	30	24	60	48	<.001**
3.	Patients who can be distracted from pain usually do not have severe pain	61	48	69	55.2	.266
4.	Patients may sleep despite severe pain	31	24.8	53	42.4	.002**
5.	Aspirin and other nonsteroidal anti-inflammatory agents are not effective analgesics for painful bone metastases	53	42.4	74	59.2	.003**
6.	Respiratory depression rarely occurs in patients who have been receiving stable doses of opioids over several months	90	72	81	64.4	.171
7.	Combining analgesics that work by different mechanisms (e.g., combining an NSAID with an opioid) may result in better pain control with fewer side effects than using a single analgesic agent	85	68	83	66.4	.790
8.	The usual duration of analgesia of 1–2 mg of morphine IV is 4–5 hours.	49	39.2	56	44.8	.260
9.	Opioids should not be used in patients with a history of substance abuse	36	28.8	33	26.4	.663
10.	Elderly patients cannot tolerate opioids for pain relief	82	65.6	90	72.0	.209
11.	Patients should be encouraged to endure as much pain as possible before using an opioid	43	34.4	49	39.2	.425
12.	Children less than 11 years old cannot reliably report pain, so clinicians should rely solely on the parent’s assessment of the child’s pain intensity	68	54.4	69	55.2	.900
13.	Patients’ spiritual beliefs may lead them to think pain and suffering are necessary	74	59.2	91	72.8	.006**
14.	After an initial dose of an opioid analgesic is given, subsequent doses should be adjusted following the individual patient’s response	105	84	104	83.2	.882
15.	Giving patients sterile water by injection (placebo) is a useful test to determine if the pain is real	30	24	23	18.4	.251
16.	Vicodin (hydrocodone 5 mg + acetaminophen 300 mg) PO is approximately equal to 5–10 mg of morphine PO	68	54.4	82	65.6	.052
17.	If the source of the patient’s pain is unknown, opioids should not be used during the pain evaluation period, as this could mask the ability to correctly diagnose the cause of pain	23	18.4	23	18.4	.053
18.	Anticonvulsant drugs such as gabapentin (Neurontin) produce optimal pain relief after a single dose	65	52	60	48.0	.595
19.	Benzodiazepines are not effective pain relievers and are rarely recommended as part of an analgesic regiment	77	61.6	82	65.6	.433
20.	Narcotic/opioid addiction is defined as a chronic neurobiological disease characterized by behaviors that include one or more of the following: impaired control over drug use, compulsive use, continued use despite harm, and craving	112	89.6	114	91.2	.657
21.	The term “equianalgesia” means approximately equal analgesia and is used when referring to the doses of various analgesics that provide approximately the same amount of pain relief	81	64.8	104	83.2	<.001**
22.	Sedation assessment is recommended during opioid pain management because excessive sedation precedes opioid-induced respiratory depression	115	92	117	93.6	.469
23.	The recommended route of administration of opioid analgesics for patients with persistent cancer-related pain is oral	50	40	34	27.2	.023*
24.	The recommended route of administration of opioid analgesics for patients with brief, severe pain from sudden onset such as trauma or postoperative pain is IV.	88	70.4	106	84.8	.003**
25.	Which of the following analgesic medications is considered the drug of choice for the treatment of prolonged moderate to severe pain for cancer patients? Morphine	105	84	109	87.2	.338
26.	A 30 mg dose of oral morphine is approximately equivalent to Morphine 10 mg IV	53	42.4	59	47.2	.408
27.	Analgesics for postoperative pain should initially be given around the clock on a fixed schedule.	89	71.2	108	86.4	.002**
28.	A patient with persistent cancer pain has been receiving daily opioid analgesics for 2 months. Yesterday, the patient was receiving morphine 200 mg/hour intravenously. Today he has been receiving 250 mg/hour intravenously. The likelihood of the patient developing clinically significant respiratory depression in the absence of new comorbidity is less than 1%.	36	28.8	30	24.0	.416
29.	The most likely reason a patient with pain would request increased doses of pain medication is related to experiencing increased pain	81	64.8	60	48.0	.008**
30.	Which of the following is useful for the treatment of cancer pain? Ibuprofen, Hydromorphone, Gabapentin, all of the above.	65	52	65	52.8	.794
31.	The most accurate judge of the intensity of the patient’s pain is the patient him/herself.	72	57.6	79	63.2	.269
32.	Which of the following describes the best approach for cultural considerations in caring for patients in pain: Patients should be individually assessed to determine cultural influences.	69	55.2	69	55.2	1.00
33.	How likely is it that patients who develop pain already have an alcohol and/or drug abuse problem? 5–15%	41	32.8	53	42.4	.115
34.	The time to peak effect for morphine given IV is 15 minutes.	87	69.6	118	89.6	<.001**
35.	The time to peak effect for morphine given orally is 1–2 hours	70	56	67	53.6	.719
36.	Following the abrupt discontinuation of opioids, physical dependence is manifested by the following: sweating, yawning, diarrhea, and agitation with patients when the opioid is abruptly discontinued	35	28	41	32.8	.398
37.	Which statement is true regarding opioid-induced respiratory depression: Obstructive sleep apnoea is an important risk factor.	54	43.2	51	40.8	.797
38a.	Patient A: Andrew is 25 years old and this is his first day following abdominal surgery. As you enter his room, he smiles at you and continues talking and joking with his visitor. Your assessment reveals the following information: BP = 120/80, HR = 80. He rates his pain as 8. On the patient’s record, you must mark his pain on the scale below. Circle the number that represents your assessment of Andrew’s pain.	35	28	36	28.8	.880
38b.	Your assessment, above, was made 2 hours after he received morphine 2 mg IV. Half-hourly pain ratings following the injection ranged from 6 to 8, and he had no clinically significant respiratory depression, sedation, or other side effects. He has: Administer morphine 3 mg IV now	10	8	9	7.2	.820
39a.	Patient B: Robert is 25 years old and this is his first day following abdominal surgery. As you enter his room, he is lying quietly in bed and grimaces as he turns in bed. Your assessment reveals the following information: BP = 120/80; HR = 80; R = 18. He rates his pain as 8. On the patient’s record, you must mark his pain on the scale below. Circle the number that represents your assessment of Robert’s pain.	50	40	50	40	1.00
39b.	Your assessment, above, was made 2 hours after he received morphine 2 mg IV. Half-hourly pain ratings following the injection ranged from 6 to 8, and he had no clinically significant respiratory depression, sedation, or other untoward side effects: administer morphine 3 mg IV now	17	13.6	16	12.8	.854

Paired sample *t*-test was used.

* *p* < .05

**p < .01

### Mean differences between pre and post the pain management education

The nurses showed moderate levels of knowledge and attitudes regarding pain pre- (M = 20.3, SD = 4.80) and post-pain management education (M = 22.2, SD = 5.09). However, their knowledge and attitudes regarding pain were significantly higher after the intervention (*t* = 2.87, *p* < .01; Table 4).

**Table 4:** Comparison of nurses’ knowledge and attitudes regarding pain scores pre and post-pain management education (*N* = 124)

Knowledge and attitudes	M (SD)	t	***p***
Post-pain management education	22.2 (5.09)	2.866	.005^*^
Pre-pain management education	20.3 (4.80)		

Paired sample *t*-test was used.

^*^p < .01

Additionally, of the sample characteristics, only nursing education level was significantly associated with knowledge and attitudes regarding pain (Table 5). Specifically, before the intervention, BSN nurses had more knowledge and attitudes regarding pain management than diploma nurses (*t* = 3.06, *p* < .01). However, there were no significant differences between the two groups after the intervention (*p* > .05), indicating the effectiveness of the intervention in enhancing nurses’ knowledge and attitudes towards pain management, regardless of their nursing education levels.

**Table 5:** Associations between the sample characteristics and their knowledge and attitudes regarding pain pre- and post-pain management education

		Knowledge and Attitudes regarding Pain					
Variable		Pre-Pain Management Education			Post Pain Management Education		
(Possible range)		(0–41)			(0–41)		
	**Range**	**M (SD)**	**t,** ***F,*** **or** ***r***	***p***	**M (SD)**	**t,** ***F,*** **or** ***r***	***p***
Sex	Male Female	21.2 (2.82) 20.2 (4.95)	0.643	.521	21.0 (5.60) 22.3 (5.04)	0.842	.401
Years of Experience	1–15		−.002	.983		−.047	.602
Level of Nursing Education	Diploma BSN	19.5 (4.22) 22.2 (5.50)	3.057	.003^*^	22.3 (5.50) 22.0 (4.12)	0.295	.768
Working Unit	Critical General Outpatient	21.4 (6.00) 19.7 (4.15) 20.9 (4.29)	1.672	.192	23.1 (5.79) 21.7 (4.65) 22.3 (5.77)	0.995	.373
Previous Pain Management Training	Yes No	20.2 (3.48) 20.5 (5.82)	0.330	.742	22.5 (5.10) 21.84 (5.11)	0.746	.457

Independent sample t-test, one-way analysis of variance, and Pearson’s correlations were used.

^*^p < .01

### Self-confidence, self-learning efficacy, and satisfaction with the intervention

Seventy-nine participants (63.2%) completed the self-confidence, self-learning efficacy, and satisfaction with learning scales after the intervention. Table 6 shows that the nurses in this study had high mean scores for self-confidence in learning (M = 35.6, SD = 4.68, range: 18–40), self-efficacy (M = 52.9, SD = 7.70, range: 25–60), and satisfaction with learning (M = 22.2, SD = 3.24, range: 10–25).

**Table 6:** Descriptive statistics for the total scores post pain management education (*N* = 79)

Variables (possible range)	Range	Mean (SD)
Self-confidence (8–40)	18–40	35.6 (4.68)
Self-learning efficacy (12–60)	25–60	52.9 (7.70)
Satisfaction (5–25)	10–25	22.2 (3.24)

## Discussion

This study evaluated nurses’ knowledge and attitudes regarding pain management before and after receiving a pain management training program. The findings revealed that the intervention significantly improved nurses’ knowledge and attitudes regarding pain management. In this study, the average KASRP score was 49.5% pre-intervention and 53.6% post-intervention, which is higher than the average of 46.25% reporting in a local multi-center study [21]. Another study conducted in Palestine [42] reported an overall mean score of 45.6%. Higher mean scores of 72% were found in Brant et al.’s (2017) study [43]. The highest mean scores were reported in a New Zealand study by Hyton (2019), with a mean score of 73.1% [44]. Nevertheless, all results fall below the recommended score of 80%, indicating that nurses worldwide have a knowledge deficit and poor attitude levels regarding pain and pain management [45]. Mędrzycka-Dąbrowska et al. (2016) elaborated on some misconceptions about pain in older adult patients that could result in inadequate pain management [9].

The overall evaluation of nurses’ knowledge and attitude revealed a good understanding of the importance of sedation assessment for patients receiving opioids for pain management. Unlike before the intervention, most nurses were knowledgeable post-intervention about the side effects and complications of opioids. A notable improvement was observed in the pain management of postoperative patients. Although the program improved nurses’ knowledge and attitude in various areas, their knowledge about the recommended opioid doses remained deficient even after the program. This deficit might be because nurses rely on physicians’ prescriptions without negotiating physician orders. Both the pre- and post-test showed that nurses believed that opioids should not be administered if the source of pain was unknown. Even after the educational program, items related to nurses’ attitudes toward the reasons patients need additional pain medication and their views that patients were the most accurate assessors of their pain were not answered correctly. Previous researchers have suggested that nurses may still have misconceptions about pain, regardless of the advancements in pain management guidelines [45].

In this study, education level was the only demographic variable significantly influencing nurses’ knowledge and attitudes regarding pain management. Specifically, nurses with bachelor’s degrees had greater knowledge and attitudes toward pain management than diploma-educated nurses. However, the knowledge levels of the two groups were comparable post-intervention, suggesting the effectiveness of the intervention. This finding contrasts with previous studies [46, 47] that reported that nurses’ educational levels were not significant factors. These inconsistent results could be attributed to differences in the samples’ demographic characteristics. More than two-thirds of the nurses in this study were diploma holders, whereas most nurses in other studies had bachelor’s or master’s degrees [46, 47]. Therefore, the current study findings highlight the importance of providing educational pain management programs for nurses with diplomas.

Nurses in this study reported high self-confidence in learning and satisfaction with what they had learned in the educational program using simulation-based experience. Similarly, Howard (2017) compared the confidence, satisfaction, and engagement levels among nursing students using simulation-based learning versus the traditional learning methods [48]. The study reported significant differences in satisfaction, self-confidence, and engagement for those learning with simulation. Students had the opportunity to give and receive feedback about their performance and observed practices in a free-risk environment. Likewise, the participants of this study had high self-efficacy scores, which is consistent with Bandura’s (1977) theory contending that knowledge is crucial for building and enhancing confidence and the ability to perform tasks [49]. Using a self-efficacy pain management scale, Alzghoul and Abdullah (2020) examined the relationship between knowledge and pain management attitudes and the perceived ability of nurses to manage pain [28]. Accordingly, they found that self-efficacy, nurses’ confidence, and the ability to manage patients’ discomfort were determined by nurses’ knowledge and attitudes, which, in turn, determined their ability to apply effective pain management techniques. These findings highlight the need to invest in rigorous educational programs that incorporate various teaching methods and techniques to enhance educational outcomes.

In this study, nurses had higher satisfaction and self-confidence regarding pain management, although their mean KASRP score remained below the recommended score of 80%, suggesting that participants may have overrated themselves. Another important aspect is that self-confidence and self-efficacy are interrelated [50]. Self-efficacy is more inclusive and has more practical implications than self-confidence [51].

### Limitations and recommendations

The current findings indicate that regular pain education programs are effective in improving nurses’ knowledge and attitudes. Future qualitative research should explore in detail misconceptions that result in unfavorable beliefs among nurses. Furthermore, knowledge should be assessed separately from attitudes to tailor education according to the assessed deficiencies. In addition to knowledge and attitude assessments, research is needed to examine the impact of educational programs and strategies, followed by clinical observational audits. Moreover, studies should consider the subjectivity and individualized nature of nursing by including patient satisfaction surveys to measure improvements in nurses’ knowledge and attitudes from the patients’ perspectives. We also recommended developing clinical guidelines for nurses to successfully implement new pain management practices [52].

This study had some limitations. First, there was no follow-up to assess retention and behavioral changes in acquired knowledge and attitudes over time. In addition, the sample used in this study was recruited using a non-random sampling method, which could adversely influence causal inferences. This study did not include a control group, which could limit the internal and external validity of the study’s findings. To overcome these limitations, future research should include randomized controlled trials to enhance the generalizability of their findings.

## Conclusion

This study examined the effectiveness of a pain management education program with nurses working in the ICU and LTC in improving their knowledge and attitudes. The intervention was beneficial in improving nurses’ pain knowledge and attitudes toward pain management. The results suggest that a well-structured education program can have a positive impact on nurses’ behaviors, influencing the quality of patient care. Regular education with clinical observation is important in ensuring that acquired knowledge is integrated into nurses’ practice.

## Data Availability

The datasets used and/or analyzed during the current study are available from the corresponding author on reasonable request.

## Abbreviations

ANOVA: One-way analysis of variance
ICU: Intensive care unit
KASRP: Knowledge and Attitudes Survey Regarding Pain
L-SES: Learning Self-Efficacy Scale
LTC: Long-term care
M: Mean
SA: Saudi Arabia
SD: Standard deviation
